# Application of Artificial Intelligence and Machine Learning Was Not Able to Reliably Predict Poor Outcomes in People With Hemophilia

**DOI:** 10.7759/cureus.66810

**Published:** 2024-08-13

**Authors:** Jianzhong Hu, Chen Lu, Bob Rogers, Martin Chandler, Jarren Santos

**Affiliations:** 1 Statistics and Data Science, American Thrombosis and Hemostasis Network, Rochester, USA; 2 Center for Digital Health Innovation, University of California at San Francisco, San Francisco, USA; 3 Statistics, SimulStat Incorporated, Solana Beach, USA

**Keywords:** ai and machine learning, clinical decision-making, predictive model, hemophilia, poor outcome

## Abstract

Background

Artificial intelligence (AI) and machine learning (ML) are currently used in the clinical field to improve the outcome predictions on disease diagnosis and prognosis. However, to date, few AI/ML applications have been reported in rare diseases, such as hemophilia. In this study, taking advantage of the ATHNdataset, an extensive repository of hemostasis and thrombosis data, we aimed to demonstrate the application of AI/ML approaches to build predictive models to identify persons with hemophilia (PwH) who are at risk of poor outcome and to inform providers in clinical decision-making towards helping patients prevent long-term complications.

Materials and methods

This project was carried out in two steps. First, the data were mined from ATHN 7, a subset study of the ATHNdataset, to determine markers that defined “poor outcome.” Second, we applied multiple AI/ML approaches on the ATHNdataset to validate our findings and to develop predictive models to identify PwH at risk of poor outcomes. The classical regression-based predictive model was used as a reference to evaluate the performance of various AI/ML models.

Results

Our models included features similarly distributed to response variables of interest, resulting in a limited ability to distinguish poor outcomes. Low recall (<53%) resulted in no single model reliably predicting poor outcomes out of all actual positive cases. Our results suggest that, to build a more useful AI/ML model, we may need a larger dataset size along with additional features. Furthermore, our results showed that most of the AI/ML models outperformed the classical logistic regression model in both model accuracy and precision.

Conclusions

Our AI and ML model showed limited ability to predict poor outcomes in people with hemophilia.

## Introduction

Hereditary bleeding disorders occur due to the absence or deficiency of specific clotting proteins or defects in platelet function. The three most common clotting protein deficiencies are hemophilia A (deficiency of factor VIII (FVIII)), hemophilia B (deficiency of factor IX (FIX)), and von Willebrand disease (vWD) [1]. The development of safe and effective hemophilia treatments has taken decades and has been primarily based on clotting factor replacement [2]. Historically, clotting factors have been given “on demand” at the time of bleeding episodes [3]. For over 20 years, prophylaxis (regular infusions of clotting factor concentrate to prevent bleeding) has been considered the standard of care in developed nations [4]. Advances in the engineering and manufacturing of clotting factor concentrates led to the widespread availability of extended half-life products, potentially reducing the number of intravenous infusions required to maintain adequate clotting factor levels [5]. The recent development of new nonfactor replacement treatments has offered therapeutic alternatives for people with hemophilia (PwH) with or without inhibitors [6]. The treatment of hemophilia is now poised for yet another transformative change: the use of gene therapy to produce functional cures in some PwH. Because these transgenes produce fully active proteins, breakthrough bleeding and the requirement for exogenous clotting factors have nearly been eliminated for most clinical trial participants [7].

Despite the tremendous advances in hemophilia therapy over the past decade, there have been multiple barriers inhibiting the uptake of new treatment technologies [8]. These barriers can exist on several levels, including those of the medical institutions, providers, and individuals with hemophilia, and include fear of the unknown and fear of failure. As factor concentrate replacement has a long track record of both safety and efficacy, and bispecific monoclonal antibodies are demonstrating both safety and efficacy, the leap to one-time therapies for conditions such as hemophilia, which is not immediately life-threatening, may be overwhelming for both providers and PwH [9]. Other impediments for PwH include short-term thinking, lack of resources, lack of adequate communication (between providers and PwH, for example), and a lack of urgency. Most important for the purposes of this project is the lack of identification at the provider level as to which PwH are at risk of a poor outcome.

Recently, artificial intelligence (AI) and machine learning (ML) approaches have been used to predict the clinical severity of hemophilia [10-13]. In this project, the American Thrombosis and Hemostasis Network (ATHN) assessed the utility of AI and ML to identify PwH who are at risk of a poor outcome with the goal of informing providers in their clinical decision-making towards helping patients prevent long-term complications. Predictive models to determine those PwH who were at high risk for developing poor outcomes were created using the ATHNdataset, an extensive repository of hemostasis and thrombosis data. The ATHN has stewardship of the ATHNdataset used in collecting and preserving this kind of data through its collaboration with over 145 treatment centers that make up the ATHN Affiliate Network. ATHN partners with these institutions to collect demographic, clinical, and genetic information and to de-identify the collected data from individuals who opt-in as part of the ATHNdataset. Data from the ATHNdataset is combined with its greater ecosystem of data and related products, ATHN Systems. The ATHNdataset is a rich data source to support clinical studies identifying, exploring, and advancing knowledge around issues affecting the inherited bleeding disorders community with the goal of transforming care.

In this study, we aimed to utilize a combination of AI and ML to identify those PwH who are at risk of a poor outcome, allowing providers to consider intensifying or altering an individual’s current therapy, including considering one-time therapy/gene therapy, with the goal of preventing long-term complications. ATHN carried out the project by accomplishing tasks centered around four objectives: to (1) develop a “poor outcome” definition; mine data from the ATHNdataset for the subset of participants enrolled in ATHN 7: Hemophilia Natural History Study to determine markers associated with poor outcomes; utilize the remainder of the ATHNdataset to validate findings from ATHN 7; and develop predictive models to identify PwH at risk of poor outcomes.

This article was previously posted to the Research Square preprint server on 18 January 2024.

## Materials and methods

Dataset

The ATHN closely collaborates with treatment centers that support the organization in its data collection efforts (ATHN Affiliates), resulting in the ATHNdataset containing data on over 23,000 PwH in the United States. The ATHNdataset represents one of ATHN’s core services for collecting de-identified data abstracted from clinical records maintained in ATHN Systems, the ATHN informatics infrastructure, from ATHN-affiliated medical sites located across the United States.

The data from ATHN 7: A Natural History Cohort Study of the Safety, Effectiveness, and Practice of Treatment for People with Hemophilia are a subset of the ATHNdataset and contain 400 participants. ATHN 7 represents ATHN’s real-world approach to assessing the safety and effectiveness of treatments used for PwH. ATHN 7 data were used as a basis to train the initial machine learning model, while the remainder of the ATHNdataset was used to test the model and validate the initial findings.

Define poor outcomes

To define a poor outcome, we conducted a survey that presented five free-text questions to determine what subject matter experts (SMEs) would report as a negative impact on quality of life: 1) Are you a patient or caregiver? 2) What is your age? 3) What is your diagnosis? 4) What do you consider to be the upsides of living with hemophilia? and 5) What do you consider to be the challenges of living with hemophilia? The survey was sent to 732 medical directors and done by the individuals with a self-reported diagnosis of hemophilia and their self-identified caregivers.

Next, we undertook a mixed-methods approach to review responses and assign categories based on SME replies. After combing the physician-defined poor outcomes, we had the initial consensus definition of SME-derived components and physician-derived components of poor outcomes.

Lastly, we filtered out components that had insufficient information in the ATHNdataset and arrived at a final consensus definition of poor outcomes, as described in the Results section

AI/ML predictive models

We employed multiple predictive models, including K-nearest neighbors (KNN) classification [14], XGBoost (XGB) [15], support vector machine (SVM) [16], logistic regression [17], gradient boosting machine (GBM) [18], random forest [19], light gradient boosting machine [20], linear discriminant analysis [21], and CatBoost [22]. The model we designed could ultimately support staff in clinical decision-making and provide an evidence-based means of identifying poor outcomes beyond the reliance on provider intuition and medical history.

## Results

Collaborating with the National Bleeding Disorders Foundation (formerly NHF) resulted in the distribution of a survey that addressed five free-text questions, as described in the Methods section, to determine what SMEs would report as a negative impact on quality of life. Among 732 SMEs invited to respond, we received responses from 64 (8.7%) over two weeks. After reviewing responses and assigning categories based on SME replies using a mixed-methods approach, we generated an initial consensus definition of SME-derived components of poor outcomes to encompass limitations in activities, arthropathy, and chronic pain. We further combined those SME-derived components with the physician-derived components of poor outcomes, including death, intracranial hemorrhage (ICH), inhibitor development, and target joint development.

As we applied our theoretical definition of poor outcome throughout the latter objectives, issues came up that prevented us from fully incorporating this first iteration. As we collected information from SMEs on patient-reported outcomes (PROs), we noticed an abundance of qualitative information that we could not capture with our present pool of information. It was beyond even the consistent PRO data we had from ATHN 7. Specifically, studies outside of ATHN 7 and ATHNdataset data did not have the same consistency and granularity on PROs compared to ATHN 7, proving impossible to evaluate a model that lacked the aforementioned information in test cases.

Though less than optimal, and for the purpose of this project only, we modified the definition of poor outcome to include one of the following (death, ICH, inhibitor development, or target joint development), paving our path to complete the other objectives using more adequate comparisons (Table 1). Although our definition of poor outcome for the purposes of this project will include the SME voice, this exercise has reinforced ATHN’s commitment to SME-centricity and inclusion in all our projects and research. We found capturing the abundance of qualitative information from SMEs invaluable in determining how the ATHN could elevate the quality of our work while capturing the SME's voice. The ATHN is developing future steps to better support collaborations incorporating diverse, inclusive, and equitable representation.

**Table 1:** Final consensus definition of poor outcomes in PwH

Final consensus definition
Death
Intracranial hemorrhage (ICH)
Inhibitor development
Target joint development

ATHN 7 possesses an extensive amount of detail on information not normally obtained in other ATHN studies and the ATHNdataset, most importantly longitudinal PROs. We first decided to use the data we gathered as part of the Patient-Reported Outcomes Measurement Information System (PROMIS®) profiles, which focused on seven domains to describe the quality of life (depression, anxiety, physical function, pain interference, fatigue, sleep disturbance, and ability to participate in social roles and activities). Data collected from the PROMIS-29 Profile aligned well with our initial definition containing components from the survey information summarized from SMEs, specifically limitations in activities, arthropathy, and chronic pain.

However, it was difficult to directly compare data from ATHN 7 to data from alternative sources, particularly the PRO data, as these data are not usually collected as part of the standard of care. Although we gathered the data necessary to address the physician- and SME-derived components outlined in our initial definition of poor outcome, we could not extrapolate the data external to ATHN 7 as it lacked the detail necessary to make adequate comparisons in an AI/ML framework. As such, we chose to move forward excluding PROs, relying on ATHN 7 sociodemographic factors (e.g., sex assigned at birth, race, education), insurance, and diagnoses at the patient level. In addition, we included vital signs and bleeds at the event level. The data elements used for training the predictive model are outlined in Table 2. We selected these variables due to the overlap existing between ATHN 7 and the ATHNdataset appropriate for model building. We did not include information, such as PROs, to test and validate the model due to the lack of detail existing in the ATHNdataset. We then cleaned and processed the data to maximize the potential of the features to be included in the model. In these steps, we grouped the level of education and employment status to reduce feature sparsity. We also adopted mean and mode imputation methods to fill in values for the missing data and applied transformations on numerical and categorical features for normalization and scaling.

**Table 2:** Data elements in ATHN 7 collected at the time of encounter used to train the predictive model

Discrete Variables	Levels (if Applicable)
Included in ATHN 7	No; Yes
Sex assigned at birth	Female; Male; Intersex
Ethnicity	Hispanic, Latino/a, or Spanish origin; Not Hispanic, Latino/a, or Spanish origin; Unknown
Gender	Feminine/woman/girl; Masculine/man/boy; Nonbinary; Transgender; Unknown
Race	American Indian/Alaska Native; Asian; Black/African American; Native Hawaiian/Other Pacific Islander White; Unknown
Education level	Elementary; College; Other
Employment status	Child; Disabled; Employed; Other
Continuous Variables	
Age; BMI; Diastolic pressure; Number of bleeds associated with a dental, medical, or surgical procedure; Number of bleeds associated with menstruation, pregnancy, or a related procedure; Number of bleeds associated with a spontaneous event; Number of bleeds associated with a trauma-related event; Number of severe bleeds; Number of unique bleeds; Pulse rate; Systolic pressure; Temperature	

Based on the final consensus definition of poor outcome, we considered a participant to experience a poor outcome if they had encountered at least one of the four physician-derived components of death, ICH, inhibitor development, or target joint development (Table 3). Our approach resulted in producing a balanced dataset of 233 participants who did not experience a poor outcome and 167 participants who did experience a poor outcome.

**Table 3:** Data elements in ATHN 7 and the ATHNdataset used to determine poor outcomes

Variable	Type	Levels (if Applicable)
Death	Discrete	No; Yes
Intracranial hemorrhage (ICH)	Discrete	No; Yes
Inhibitor development	Discrete	No; Yes
Target joint development	Discrete	No; Yes

**Figure 1:**
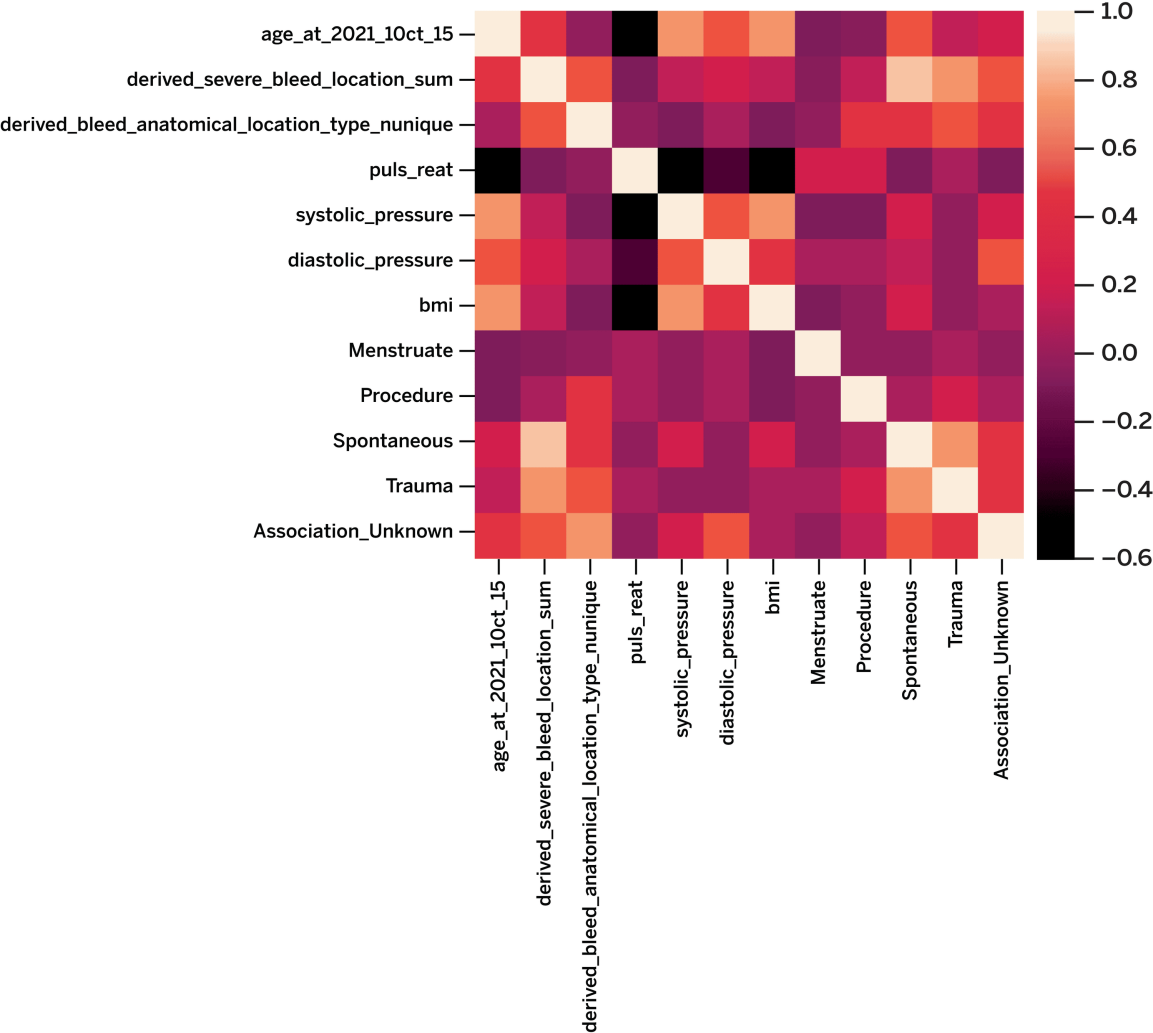
Heatmap of the correlation between continuous features used in the model

The team first conducted exploratory data analysis by carrying out tasks such as determining the distributions of each variable and assessing what variables could be correlated with poor outcomes (Figure 1). We then merged data across ATHN Systems to preprocess and generate the final dataset used for predictive modeling. Afterwards, we split the data into sets with 75% of the ATHN 7 data used for training and the remaining 25% used for testing. Finally, we cross-validated the predictive model with the ATHNdataset data using grid search and k-fold stratification to determine accuracy and predictive power.

To make use of AI and ML in clinical outcomes research, we applied multiple AI/ML classifier algorithms including KNN classification, decision trees, and random forests classifiers. Logistic regression was used as the benchmark reference. We pivoted to using other outcomes such as accuracy, precision rate, and recall rate that allow us to immediately evaluate accuracy given our setup of poor outcome prediction. Accuracy is calculated by obtaining the correct number of predictions out of all predictions. Precision is calculated by obtaining the correct number of predictions of participants who do experience poor outcomes out of all predictions of participants who do experience poor outcomes. Recall is calculated by obtaining the correct number of predictions of participants who do experience poor outcomes out of the correct predictions of participants who do experience poor outcomes and the incorrect predictions of participants who do not experience poor outcomes.

The baseline model results yielded the following results where the accuracy range is based on the fourfold cross-validation (Table 4). The KNN model achieved the highest accuracy of 61.67% with the broadest range of accuracy, while the decision tree model achieved the lowest accuracy of 56.33% with an average range of accuracy. The RF model achieved a similar accuracy of 61.33% to that of the KNN model with the narrowest range of accuracy.

**Table 4:** Initial baseline model results using ATHN 7 to train and the ATHNdataset to test/validate findings

Model Used	Accuracy	Accuracy Range
K-Nearest Neighbors (KNN)	61.67%	(56.67%, 66.67%)
Random Forest (RF)	61.33%	(58.67%, 64.00%)
Logistic Regression	61.33%	(57.33%, 65.33%)
Decision Tree	56.33%	(53.33%, 59.33%)

Because the baseline model results did not meet our expectations, we sought to improve model accuracy. As mentioned in previous sections, we no longer considered PROs as valid features in building the predictive model given that the remainder of the ATHNdataset did not meet the same level of detail as ATHN 7. This decision led us to believe that no distinguishable difference between ATHN 7 and the ATHNdataset seemed to exist, aside from increased missingness in the ATHNdataset, moving the team to split the data differently. We incorporated more ATHNdataset data in the training set and more ATHN7 data in the testing set. We believed this would help us obtain potentially higher predictive power by supplying the training set with more data than just the original ~400 data points.

We extended the dataset and removed participants with missing values in the numerical variables, which led to 12,414 total participants with 9,664 who did not experience a poor outcome and 2,750 who did experience a poor outcome. After making this adjustment, we repeated the process of conducting exploratory data analysis, merging, data splitting, and k-fold cross-validation to determine the accuracy and predictive power while applying the same transformations and encoding methods in the baseline model. Additionally, because of the larger, maximally transformed dataset, we modified our previous predictive modeling approach and implemented nine different classification algorithms on the ATHNdataset data. However, the ATHNdataset data did not achieve the same performance as we found in the ATHN 7 data.

**Figure 2:**
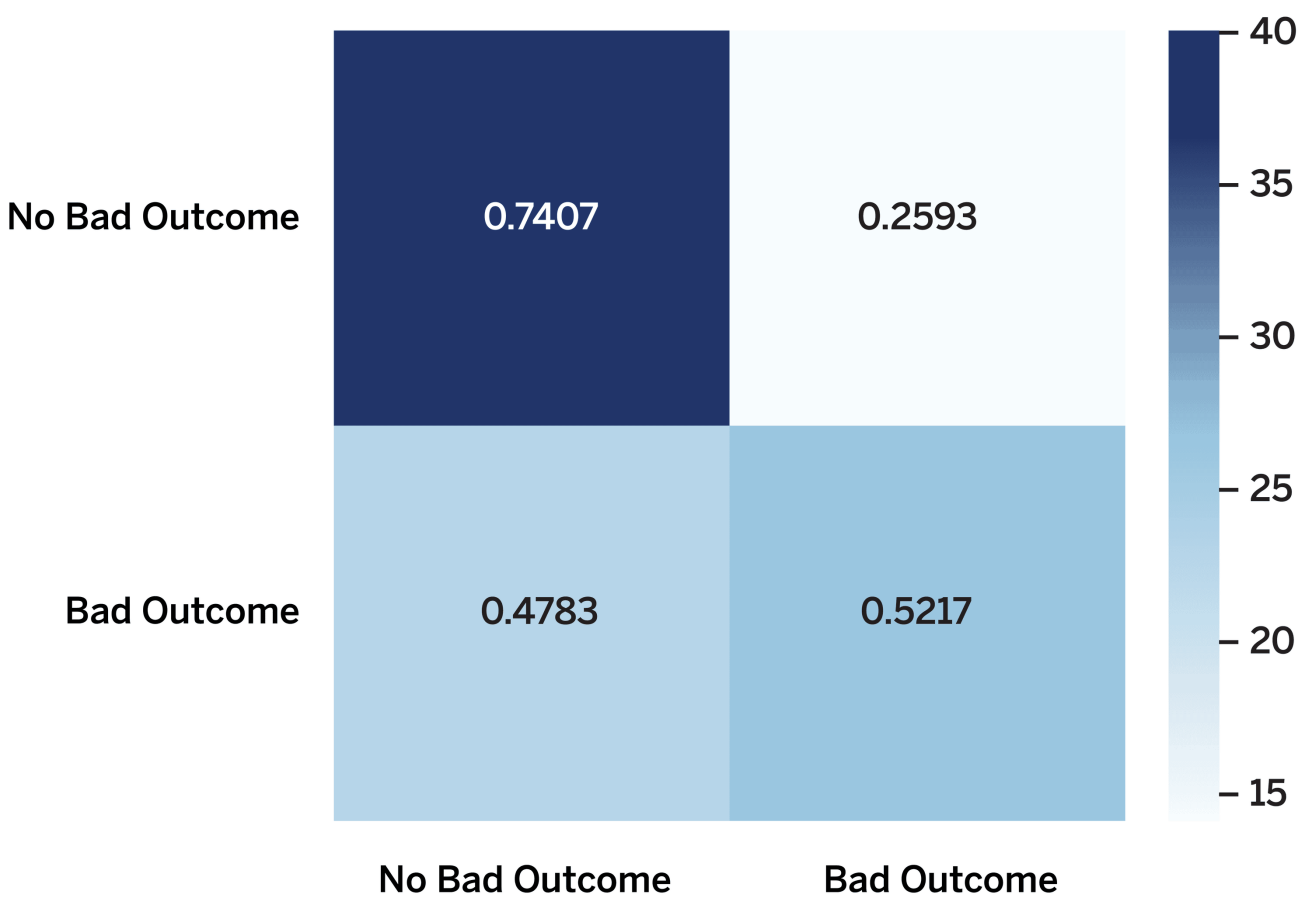
Confusion matrix of the XGBoost model in predicting poor outcomes in the transformed dataset out of 100 participants *A darker color represents more participants, whereas a lighter color represents less.

We also applied the modified predictive modeling approach to the ATHN 7 data, which yielded the following results (Table 5). Overall, the CatBoost model obtained the highest accuracy of 67.00% and the highest precision of 80.95%. The XGB model obtained the highest recall of 52.17% while achieving similar accuracy to that of the CatBoost model. Across all classification models, we observed low performance in recall. The algorithms had difficulty finding signals to declare if a patient was likely to experience poor outcomes with the features included in the training data. For example, the confusion matrix of the XGB model showed sensible accuracy for predicting those who did not experience a poor outcome, but inability to distinguish those who did experience a poor outcome (Figure 2).

**Table 5:** Modified model results combining ATHN 7 and the ATHNdataset into a larger, maximally transformed dataset

Model Used	Accuracy	Precision	Recall
CatBoost	67.00%	80.95%	36.96%
XGBoost (XGB)	64.00%	63.16%	52.17%
Support Vector Machine (SVM)	63.00%	66.67%	39.13%
Light Gradient Boosting Machine (LGBM)	62.00%	60.53%	50.00%
Random Forest (RF)	62.00%	61.76%	45.65%
Gradient Boosting Machine (GBM)	60.00%	57.50%	50.00%
K-Nearest Neighbors (KNN)	57.00%	54.55%	39.13%
Logistic Regression	54.00%	50.00%	30.43%
Linear Discriminant Analysis (LDA)	51.00%	45.16%	30.43%

We obtained mixed results in our modified predictive modeling approach; some models achieved relatively high performance (>75%) in overall accuracy, but the majority failed to meet an adequate recall rate. This raised the idea of applying what we had done here to certain components of the final consensus definition of poor outcomes. Some noise may exist across the entirety of the dataset due to a variety of reasons, particularly the imbalance in the distributions of some features and outcomes. Among those four physician-derived components of death, ICH, inhibitor development, or target joint development, the target joint has a balanced split across the transformed dataset.

Therefore, we chose to take a deeper dive into investigating how our approach may predict the outcome of target joint development.

Our application of the modified predictive modeling approach to target joint development as an outcome yielded the following results (Table 6). The RF model achieved the highest overall accuracy of 79.00% and the second-highest precision rate of 61.11%. The CatBoost model achieved the highest precision rate of 70.59% with a similar accuracy of 75.00% to that of the RF model. Similar to what we examined in the modified predictive modeling approach to poor outcome, we observed a low recall rate throughout all classification models with none reaching 50.00%.

**Table 6:** Modified model results looking only at the target joint development as a component of poor outcomes

Model Used	Accuracy	Precision	Recall
Random Forest (RF)	79.00%	61.11%	44.00%
K-Nearest Neighbors (kNN)	78.00%	57.14%	48.00%
Gradient Boosting Machine (GBM)	77.00%	54.55%	48.00%
XGBoost (XGB)	77.00%	54.55%	48.00%
Light Gradient Boosting Machine (LightGBM)	76.00%	52.63%	40.00%
CatBoost	75.00%	70.59%	37.50%
Logistic Regression	75.00%	50.00%	36.00%
Linear Discriminant Analysis (LDA)	73.00%	45.45%	40.00%
Support Vector Machine (SVM)	73.00%	45.00%	36.00%

The modified predictive modeling approach applied to poor outcomes performed similarly when applied to target joint development. Models achieved decent overall accuracy while failing to distinguish who experienced a poor outcome between those who truly experienced a poor outcome and those who did not. We believe that the noise from the ATHNdataset participants who lack complete information on the features included quintessentially explains the low recall rate. However, the imbalance in some variables and the overall lack of signal in the data may also contribute to these findings.

## Discussion

To determine a consensus definition of “poor outcome” in PwH, physicians at ATHN-affiliated centers and SMEs were surveyed. For physicians, the options for poor outcomes were limited to those defined by the European Haemophilia Safety Surveillance (EUHASS). For SMEs, the survey was designed to allow free-text answers; therefore, a qualitative analysis approach was taken to assess the responses. Based on these methodologies, the physician-derived and SME-derived consensus definitions of poor outcomes were selected. This approach to the development of a consensus definition of poor outcomes in PwH has several strengths. First, all physicians at ATHN-affiliated treatment centers in the United States were given the opportunity to respond. Although the response rate was low and limited to pediatric providers, there was overwhelming consensus as to the definition of poor outcomes. Second, this was the first collaboration between ATHN and the National Bleeding Disorders Foundation in utilizing conversion rate (CVR) to directly query SMEs and incorporate the voice of those who experience hemophilia for their input into an ATHN project.

Although there were similarities in the themes of the outcomes identified by the two groups, there was seemingly a lack of overlap in the precise events defined as poor outcomes. This lack of accord between the physicians and SMEs could be due to issues related to how the data were collected, as well as the respondents’ differing perspectives. First, the choices of poor outcomes presented to physicians were prescriptive in nature as we wanted to ensure our ability to harmonize our definition of poor outcomes with data collected within ATHN Systems and EUHASS. For SMEs, we were interested in obtaining a more holistic understanding of the daily challenges associated with living with hemophilia. In addition, despite several revisions by multiple people, asking about the “challenges of living with hemophilia” may not have completely harmonized with the question to physicians with a limited number of poor outcomes from which to choose. That said, as physicians and SMEs do not have or use the same vocabulary, what appears to be a discordance, may, in truth, reflect the ability of respondents in the two populations to communicate equivalent ideas using different vernacular. For example, the physicians’ choice of “target joint development” may represent the SMEs’ identification of “chronic pain” and “limitation in activity” as physicians approached this task from a biological perspective and SMEs approached the task from a psychosocial perspective.

Our approach to arriving at this consensus definition of poor outcome did have several limitations. The response rate for both physicians and SMEs was low. The physicians were all pediatric providers, potentially affecting the generalizability of the responses as no input from non-pediatric hematologists was obtained. In addition, there was no potential for the physician respondents to add alternative choices of potentially poor outcomes to the list of choices they were provided. Finally, we did not get input from other multi-disciplinary treatment center team members, limiting the diversity of input as well as the diversity of background and experience of the respondents.

While time efficient, utilizing CVR as a mechanism to obtain input from SMEs, compared to going through ATHN-affiliated centers or offering a public survey through social media, may have negatively impacted our ability to recruit higher numbers of SME respondents. Next, neither the physician nor SME surveys were open for long periods of time, leading to limitations in recruitment and scope. Furthermore, we did not collect demographic information such as sex assigned at birth, gender, race, and ethnicity, from either the physician or SME respondent population, impacting our ability to assess the inclusion of an appropriately diverse population of respondents from either group. In addition, as both data acquisition approaches required the ability to access and use the internet, we missed collecting input from those individuals who lack such access, are not savvy in the use of technology, or choose not to share opinions in such forums. Finally, the confidence levels of our AI/ML models are low and the models were not further validated in another population.

Despite the limitations, this is the first consensus opinion as to the definition of poor outcomes in PwH based on perspectives from both physicians and SMEs. ATHN will use these definitions as a proof-of-concept in the next AI and ML project. If these definitions lead to a predictive model of poor outcomes, we will be able to take a more inclusive approach to determining a final definition of poor outcomes for PwH.

We attempted to develop a predictive model to determine those PwH who were at high risk for developing our predefined poor outcome using AI and ML algorithms. As we conducted exploratory data analysis, options for predictive models were discussed; different feature sets were tested to determine which models to apply and how certain features should be added to the training data. We were expecting to predict poor outcomes well with participant sociodemographic data and vital readings (e.g., age, diastolic pressure), as well as derived features from metrics collected at visits and blood events (e.g., BMI, number of specific bleeds). However, we noticed that most of the included feature variables have a very similar distribution among subsets by the response variables that we wanted to predict, which meant features would be less likely to contribute to the distinction of the response variables. We observed this in the classification results from the baseline model. Therefore, the data we had available to “teach” our algorithm to learn a pattern was not enough. As previously discussed, the data in the ATHNdataset had a high level of missingness and little in the way of PROs to help guide this machine learning. In addition, ATHN 7 did not have enough longitudinal data to ensure adequate learning. Although the results were laudatory, the data available in the ATHNdataset and/or ATHN 7 were lacking enough patterns to come to a meaningful predictive model.

## Conclusions

Without the ability to develop a model with a high recall rate, our model would not be appropriate for clinical decision-making with regard to predicting poor outcomes with our available data. However, after applying the more complex boosting models such as CatBoost and XGBoost, we do see some improvement in the performance, which means that there may be an opportunity to improve the predictive power if we are able to obtain more, higher-quality clinical data related to hemophilia. As ATHN improves its data collection to account for a more extensive longitudinal set of data, including an ever-increasing number of PROs as well as genotypic data around factor VIII or IX variants and the associated genome-wide association studies (GWAS) utilizing data from the My Life, Our Future project, and the associated Trans-Omics for Precision Medicine (TOPMed) data, ATHN’s ability to refine a model for poor outcome will be enhanced.
